# Promising Therapeutic Functions of Bone Marrow Mesenchymal Stem Cells Derived-Exosome in Asthma

**DOI:** 10.1155/2022/1485719

**Published:** 2022-12-20

**Authors:** Jia-Ying Yuan, Xiang-Yun Wang, Zhi-Ying Tong, Yu-Chao Dong, Jia-Yi Zhao, Yi Zhang, Yan Shang

**Affiliations:** ^1^Department of Respiratory and Critical Care Medicine, The First Affiliated Hospital of Naval Medical University (Second Military Medical University), Shanghai 200433, China; ^2^Department of Respiratory Medicine, Kongjiang Hospital, Yangpu District, Shanghai 200093, China; ^3^Department of General Medicine, The First Affiliated Hospital of Naval Medical University (Second Military Medical University), Shanghai 200433, China

## Abstract

Asthma is a chronic inflammatory disturbance of the airways in which many cells and cellular elements are involved. Wheezing, breathlessness, chest tightness, and coughing, especially at night or in the early morning, are typical symptoms of asthma. At present, inhaled corticosteroid (ICS) and long-acting *β*-agonists (LABAs) are standard treatments for regular management. Oral corticosteroids (OCSs) were recommended for controlling asthma exacerbation but only for a short-term treatment because of the side effects on organs. Biologic therapies have achieved exciting and notable effects in clinical treatment but are not applicable for all phenotypes of asthma. At present, some new approaches are under exploration to lessen side effects and improve curative effects. Studies have revealed that bone marrow mesenchymal stem cells (BMMSCs) hold various curative effects in asthma and may benefit in the long term with high safety. Extracellular vesicles (EVs) enriched in body fluid were characterized as subcomponents of extracellular vesicles and delivered carriers combined with genetic messages in vivo. The therapeutic potential of exosomes has become a research hotspot in many diseases. BMMSC-derived exosomes were considered as the dominant part of BMMSCs in cell-to-cell communications and playing curative effects. Points also hold that BMMSC-Exo could interfere with airway inflammation and airway remolding in asthma via modulating the immune response, regulating gene expression, adjusting the phenotype of macrophage, etc. However, BMMSC-Exo still lacked more clinical trials for evaluating the effects on asthma, and the technology of extraction and purification still needs to be improved for wide use. This review aims to draw the relationship among asthma, BMMSC, and exosome, which may provide innovate ideas for treatment of asthma, and arouse attention about the curative potential of BMMSC-Exo.

## 1. Introduction

Asthma is a heterogeneous disease with various pathogenesis and clinical features, influenced by epigenetic regulation and environmental effects. Meanwhile, asthma has some similar symptoms, including wheezing, dyspnea, chest tightness, and cough [1]. There are approximately 300 million asthma patients worldwide, and the prevalence of asthma is still growing, which poses a substantial burden to society, especially in low-income countries [2]. In China, with the development of urbanization and industry, the number of asthma patients has been increasing fast over the past decade [3]. Asthma can originally occur at any age, even in the elderly [4]. Interestingly, asthma is more common in boys than girls, while the morbidity of asthma in adult women is much higher than that in adult men [5].

According to the research that aims to identify asthma phenotypes, the Severe Asthma Research Program (SARP) in the United States found five phenotypes of asthma: (1) mild early-onset allergic disease, (2) moderate early-onset allergic disease, (3) late-onset eosinophilic nonallergic disease, (4) severe early-onset eosinophilic allergic disease, and (5) late-onset nonallergic neutrophilic severe asthma with fixed airflow obstruction [6]. Five groups of severe asthma were identified via SARP based on the onset age, allergen stimulation, lung function, medical treatment, healthcare, and comorbidities [7]. Allergic asthma was the most common type of asthma. Besides, four distinct subtypes of asthma were proposed that were based on the inflammatory cell count in induced sputum: eosinophilic asthma (eosinophils > 1.9%–3%), mixed eosinophilic and neutrophilic asthma, neutrophilic asthma (neutrophils > 61% and total cell count greater than 10 million cells/g), and paucigranulocytic asthma (neutrophils and eosinophils both within normal range) [8]. The endotypes of severe asthma were usually categorized as type 2 high severe asthma, type 2 low severe asthma, and mixed [9].

Clinical history and examination are basic and indispensable ways for diagnosis. Lung function testing, allergy testing, and the measurement of exhaled nitric oxide levels are commonly used in clinical diagnosis [10]. Besides, peak expiratory flow rate (PEFR) can show diurnal variable airway obstruction to help diagnose and monitor asthma. Pre- and post-bronchodilator spirometry play an important role in assessing reversibility. It was considered that the forced expiratory volume in 1 second (FEV1) increasing over 12% and 200 mL can be a hint of asthma, while increasing over 400 mL will be strong evidence of asthma [11]. A 3-year prospective analysis revealed the asthma exacerbation was highly relevant to the rapid decline of lung function despite regular therapy with ICS, and variable FEV1 seemed more common in the elderly and in people who have higher levels of FENO [12]. Asthma in child could make substantial adverse influence both in physic and mental, asthma also may cause approximately 13.8 million absent school days in USA in 2013; besides, children with severe asthma had about twice costs in medical care than those with wild or moderate asthmatic children in long-term life [13]. A large-scale historical prospective study in Japan found that severe and uncontrolled asthma adult patients had more medical and economic burdens than severe and controlled asthma patients and moderate asthma patients; thus, well management of asthma patients and finding appropriate treatment in the clinic seemed extremely essential [14].

## 2. Advances in Research on Asthma

### 2.1. The Pathology of Asthma

Airway inflammation was considered as an important part in asthma progression. There is heterogeneity in airway inflammation in asthma, in which subtypes could be identified via the eosinophil (EOS) and neutrophil (NE) proportions [15]. Mast cells and eosinophils combined with IgE (immune globulin E) were considered to initiate inflammation in allergic asthma [16]. EOS also could activate the expression of several proinflammatory cytokines, chemokine, growth factors, and lipid mediators to break lung homeostasis, and induce airway inflammation in asthma; and the imbalanced levels of distinct subtypes of EOS may promote the asthma progress, which provides a novel individualized treating approach for asthma [17]. EOS also could regulate immune response in asthma, modulate Th2 cell and M2 macrophage polarization, promote ILC2 accumulation, and upregulate the expression of IL-5 and IL-13 which mainly induced airway remodeling [18–20]. Neutrophils were identified to release certain nuclear proteins and serine proteases which participate in the formation of neutrophil extracellular traps (NETs), and NETs may activate the inflammasome in monocytes or macrophages to upregulate the expression of several inflammation cytokines [21].

The nuclear factor kappa-B (NF-*κ*B) signaling pathway was commonly regarded as a basic initiator and regulator in airway inflammation; proinflammatory factors, viruses, drugs, cigarette smoke, activators of protein kinase C (PKC), and many other stimulations could act as initiators in NF-*κ*B activation. Along with ubiquitination of phosphorylated inhibitory *κ*B proteins (I*κ*Bs) and proteasome-dependent proteolysis, NF-*κ*B transposition to the nucleus to promote inflammation [22]. In the cellular and mouse model of asthma, lipopolysaccharide (LPS) could combine with toll-like receptor 4 (TLR4) to activate myeloid differentiation primary response 88 (MyD88), and the nuclear transposition of NF-*κ*B happened then. Besides, high mobility group box 1 (HMGB1) secreted by some injury cells or immune cells in asthma could promote the activation of TLR4, inhibited by heat shock factor 1 (HSF1), when bound with HMGB1 promotor [23, 24]. In TH2-associated asthma, NF-*κ*B was found to be targeted by TLR4 to promote asthma exacerbation [25]. NF-*κ*B acted as an important mediator in immune-inflammatory responses and also could be activated by angiotensin II to enhance the expression of TNF-*α*, CD40, IL-6, etc. [26].

Notably, airway remodeling was a key consequence of chronic airway inflammation, which promotes airway hyperresponsiveness (AHR) and lung function limitation [27]. While some researches hold that airway remodeling and airway inflammation seemed to evolve in parallel; airway remodeling also could be regulated by some changes of structural cells and inflammatory factors [28]. Airway epithelial damage, mucous gland hyperplasia, subepithelial basement membrane deposition of collagen, and ASMC proliferation are considered as some of the main remodeling changes [29]. Pavan et al. pointed that airway smooth muscle (ASM) accumulation was related to inducing contractile phenotype, and the increasement of laminin *α*4 and *α*5 in ASM may promote the expression of EOS and ASM mass [30]. Besides, epithelium damage also played an essential role in airway remodeling, with promoting mucus hypersecretion, inducing subepithelial fibrosis, and worsening airway shortening [31].

**Figure 1:**
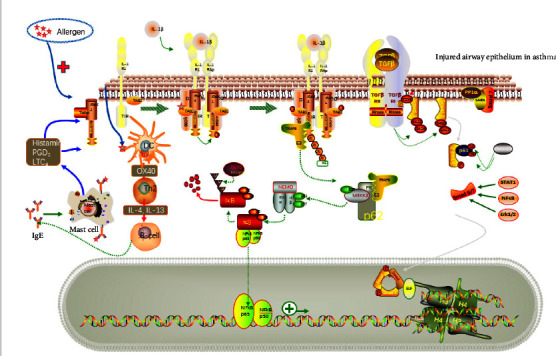
Molecular pathological mechanisms of asthma.

Complex signaling pathways and factors were involved in airway remodeling. It was revealed that transforming growth factor-*β*1 (TGF-*β*1) and signal transducers and activators of transcription (STAT) 3 were stimulated by the NF-*κ*B pathway in inducing airway inflammation and could be inhibited by interleukin (IL-37) in alleviating airway smooth muscle cell (ASMC) proliferation [32]. TGF-*β*1 mainly derives bronchial epithelial cells and eosinophils, upregulating the expression of fibroblasts, regulatory T cells, and CD8+ T cells and inhibiting differentiation of TH1 and TH2 cells so as to aggravate airway hyperresponsiveness and inflammation in asthma [33]. Indeed, the TGF-*β* family owns three isoforms as TGF-*β* 1, 2, 3; studies have conveyed that TGF-*β*2 was as important as TGF-*β*1 in regulating airway inflammation and promoting subepithelial collagen deposition in asthma [34]. The progress of airway remolding was also enhanced by wnt/*β*-catenin signaling pathway, in which wnt/*β*-catenin may mediate the p38 MAPK signaling pathway via c-Myc and cyclin D1 as two target factors [35]. Wu et al. revelated that the lack of phosphatase and tensin homologue deleted on chromosome ten (PTEN) could promote the ASMC proliferation and migration, and induce airway remodeling in asthma; the inhibition of the TNF-*α* stimulation and the cluster of differentiation 38 (CD38)-mediated Ca2+/cyclic AMP response-element binding protein (CREB) signaling mediated by PTEN may act as the potential molecular mechanism in that [36] (Figure 1).

### 2.2. Some Novel Treatments of Asthma

At present, there are varied treatments for asthma. According to the GINA and the Bronchial Asthma Guidelines, ICS was recommended as a first-line drug in treating asthma. Using ICS rationally, combined with monitoring peak flow, is regarded as helpful in asthma control [37]. ICS makes an active effect in controlling asthma symptoms to reduce inflammation and airway hyperresponsiveness [38]. As the stepwise approaches for adult asthma patients management advised, regular low-dose ICS and SABA as-needed were preferred for mild asthma, ICS-formoterol were recommended for moderate asthma patients based on numerous clinical evidences, and severe asthma patients were recommended ICS-LABA plus LAMA or OCS [39], while the LABA should be avoided for use as monotherapy [40]. OCS was essential for uncontrolled severe asthma patients and asthma exacerbations, while OCS exposure increased the risk of OCS-related complications at the same time and influenced many organs such as bone, muscle, skin, and so on [41].

At present, biologic therapy expressed significant effect in asthma management and glucocorticoid-sparing. Omalizumab, a humanized monoclonal antibody against IgE, could inhibit Th2 inflammation and reduce eosinophil counts in blood which expressed active function in adolescent [42]. In addition, a clinical study of omalizumab in Japan proved that omalizumab obviously reduced allergic asthma recurrence and exacerbation, contributed to the severe allergic asthma management, and the old with asthma also benefits a lot; while the specific effect and application of omalizumab in nonallergic asthma or other types of asthma are not clear at present [29].

Benralizumab (IgG1-kappa humanized monoclonal antibody against IL-5R*α*), dupilumab (IgG4 human monoclonal antibody against IL-4R*α*), mepolizumab (IgG1-kappa humanized monoclonal antibody against IL-5), and reslizumab (IgG4-kappa human monoclonal antibody against IL-5) have been used in asthma patients and proved to play active and hopeful treating effects in some asthma patients; while the efficacy is not very certain because of the asthma heterogeneity and may be influenced by baseline asthma severity, lung function, exacerbation history, and asthma duration, and individualized precision treatment may be mainly demanded [43–45]. Mepolizumab, reslizumab, and benralizumab were three monoclonal antibodies that focused on reducing the expression of EOS. One clinical trial found the lung function and life quality showed no significant difference, while the EOS was obviously reduced by the use of mepolizumab [46]. Notably, more points insisted that the use of anti-EOS antibodies could help reduce the risk of severe exacerbations, decrease the dose of OCS, improve lung function, and long-term life quality [47]. However, those biologic therapies mostly focused on inhibiting type-2 airway inflammation in asthma, while the patients without a Th2-high endotype did not get much benefit from those biologic therapies [48]. Interestingly, tezepelumab, a novel therapy, is a human monoclonal antibody (IgG2*λ*) that could specifically bind to thymic stromal lymphopoietin (TSLP). In the phase IIb PATHWAY study lasting for 52 weeks, tezepelumab was well tolerated, reduced the asthma exacerbation by about 55–83% when compared to the placebo group, decreased the expression of multiple Th2 inflammatory mediators, and showed more treatment effects with less correlation to baseline Th2 biomarker profiles in severe asthma [49].

As comorbid asthma-panic disorder (PD) occurs at a high rate in the population, it was reported that cognitive behavior psychophysiological therapy (CBPT) and music and relaxation therapy (MRT) both showed advantages in obviously reducing respiration rate, blood pressure, heart rate, and anxiety to help improve asthma control, in which CBPT was superior to MRT in improving the adherence to ICS [50]. Furthermore, group cognitive behavioral therapy (GCBT) might modulate the autonomic nervous system and supplementary motor area (SMA) to adjust breathing, encourage asthmatic patients to overcome their worry and panic, and recover abnormal functional connectivity (FC) for better symptom control [51].

TCM, which was characterized as an individualized and multiplex meridian intervention, expressed promising effects in improving pulmonary function and alleviating asthma [52]. A survey conducted by the American Academy of Allergy, Asthma, and Immunology found that more than 50% of patient members received traditional Chinese medicine (TCM) treatments; additionally, ASHMI, a herbal medicine, was approved by the Food and Drug Administration for clinical evaluation [53]. In the mouse models of asthma, scorpio and centipede (SC), as two kinds of insect, Chinese medicine not only showed anti-inflammatory effects in treating asthma but also played an important role in modulating the expression of exosomal microRNAs (miRNAs) in asthma through regulating some key signal pathways in asthma such as Wnt and MAPK pathways [54]. Yingying et al. found that the TCM herbs helps in tonifying Qi and kidney, and replenishing spleen could reduce the number of asthma attacks, improve clinical symptoms, and lessen airway resistance in asthmatic children aged 2 to 5 years old [55]. Summer acupoint application treatment (SAAT) was also widely used in clinics for its significant curative effect and low cost. A report showed that SAAT can stimulate a function-specific point to decrease cellular inflammation and the frequency of asthma exacerbations [56]. However, the clinical research on TCM with satisfactory curative effect is inadequate, and the mechanisms remain unclear, so more efforts should be made for its clinical application worldwide.

## 3. Exosomes and Their Specific Effects in Asthma

### 3.1. The Biology of Exosome

EVs were regarded as secreted membrane-enclosed vesicles which own bilayer-enclosed lipid [57]. Exosomes belong to the collection of EVs, and their formation began with the microautophagy of late endosomes [58]. When the endosome finished sprouting inward, multivesicular bodies (MVBs), which contained nanovesicles, were triggered to move to the plasma membrane and fuse with it; and after the fusion, the nanovesicles in the MVBs were released and were renamed to exosomes (30–150 nm in diameter) [59, 60]. Besides, LBPA (or bis(monoacylglycero)phosphate) was found to be enriched in late endosomes, which plays a vital role in promoting the formation of exosomes [61].

Exosomes exist in most body fluids with some certain proteins and nucleic acids included. It is observed that the morphology of exosomes is various when derived from different bodily fluids, indicating the functions of exosomes may also be diverse [62]. The study on urinary tract infections (UTIs) demonstrated that urinary exosomes could load certain molecules to attract bacteria and act as vehicles for antimicrobial molecules [63]. In the study of inflammatory bowel disease (IBD), stool-derived EVs were identified as playing an important role in balancing the immune homeostasis in the gut and may become novel biomarkers and targets for treating IBD [64].

In addition, exosomes were characterized as carrying rich cargos, such as proteins, RNAs, lipids, and so on, and those cargos can be transferred into cells nearby to influence the cells' functions [65]. For instance, AF-derived exosomes (AF-Exos) were considered loaded with abundant growth factors. Due to these features, AF-Exos expressed special abilities in restoring injured testicular tissue and inhibiting cell apoptosis to increase regeneration in the rat model [66]. Besides, some of the proinflammatory proteins were identified to be encountered in respiratory exosomes, such as LCN2, S100A12, Serpin A, APOA4, and so on, which may influence the oxidative damage and modulate the inflammatory progress [67]. Exosome-derived Multiple Allogeneic Protein Paracrine Signaling (Exo-d-MAPPS) was extracted from placental tissues by MSC-Exo, which could promote lung repair and regeneration, deliver anti-inflammatory factors, and expand immunosuppressive cells in lung for improving lung function and alleviating inflammation [68]. In chronic obstructive pulmonary disease (COPD) patients, miR-21 were investigated to be overexpressed in exosome derived from bronchial epithelial cells and could be transferred from bronchial epithelial cells to bronchial fibroblast cells in inducing myofibroblast differentiation [69]. Furthermore, Gupta et al. pointed out that airway epithelial-derived exosomal cargo may directly target recipient cells and impact protein expression, modulate mucus hypersecretion, and influence airway remodeling in chronic lung disease [70]. In the model of acute lung injury, human endothelial progenitor cells (EPCs) were proven to transfer miR-126 into lung epithelial cells to enhance the expression of tight junction proteins and target HMGB1 and VCAM1 expression to reduce inflammatory cytokines and lung epithelial dysfunction [71].

### 3.2. Exosome in Asthma

The expression of exosomes can be detected differently in the bronchoalveolar lavage fluid (BALF) of asthma patients [72]. Exosomes may have the potential to regulate the progress of asthma. Indeed, most of the key cells in the progression of asthma, such as mast cells, eosinophils, dendritic cells (DCs), T cells, and so on, were identified to release exosomes, showing different functions and influencing the communications between cells [73].

As a result of the different sources of exosomes, exosomes always vary in function. Hough et al. found that several lipids of EVs in BALF, such as ceramide, sphingomyelin, and leukotrienes, expressed obviously different patterns between asthma patients and healthy subjects, and those changes may promote the inflammatory progress in asthma [74]. In addition, certain surface molecules on MHC-classII-selected exosomes, such as CD36, were identified as upregulated in asthma patients to take part in airway inflammation, while the BALF exosomes were also considered to enhance the expression of leukotrienes B4 (LTB4) and LTA4H to induce asthma inflammation [72]. Besides, the B cell-derived exosome may produce allergen-derived peptides and activate allergen-specific T cells directly in allergic diseases [75]. Rat alveolar macrophages could secret exosomes which contained high levels of miR-21-5p and transferred miR-21-5p to tracheal epithelial cells to induce EMT and promote remodeling progression via activating TGF-*β*/Smad signaling pathway [76]. The release of neutrophils-derived exosomes could increase ASM proliferation and extracellular matrix remodeling in asthma [77]. In addition, eosinophil-derived exosomes may accelerate cell apoptosis of small airway epithelial cells (SAECs), enhance proinflammatory gene expression, such as TNF, CCL26, and POSTN, and trigger the MAPK cascade to promote bronchial smooth muscle cells (BSMCs) proliferation [78].

However, exosomes also showed notable beneficial effects. In the treatment of scorpio and centipede (SC) in asthma, BALF-derived exosomal miR-98-5p and miR-10a-5p were found to increase and evolve in reducing AHR, airway inflammation, and regulating Wnt and MAPK pathways [54]. In addition, M2 macrophage exosomes could obviously suppress the expression of proinflammatory factors such as IL-1 and transfer miR-30b-5p to modulate airway epithelial cell pyroptosis for treating [79]. MSC-exosomes could increase the ratio of IMs in the lung and promote the production of IL-10 to combat ovalbumin (OVA)-induced allergic asthma [80].

Interestingly, some functional MVBs have been found to secrete exosomes from eosinophils of asthmatic patients, in which exosomes may act as a biomarker in asthma [81]. Vázquez-Mera et al. recently proved five T cell-specific miRNAs: miR-21-5p, miR-126-3p, miR-146a-5p, and miR-215-5p, which were recommended with high confidence to act as noninvasive biomarkers in clinical use for identifying the phenotype, endotype, and severity of asthma [82].

## 4. Bone Marrow Mesenchymal Stem Cells-Derived Exosome (Table 1)

### 4.1. Bone Marrow Mesenchymal Stem Cells and Asthma

Nowadays, cell-based therapy seems to become a study spot and may become a novel treating approach. Mesenchymal stem cells (MSCs) are considered to have the capacity for self-renewal and exist in adipose tissue, umbilical cord blood, bone marrow, and so on [83]. BMMSC was isolated from bone marrow, which was widely used for its abundant source and easy availability, showing therapeutic potential in many immune and inflammatory diseases, such as COVID-19, acute refractory graft-vs-host disease (GVHD), chronic obstructive pulmonary disease (COPD), and asthma [84]. BMMSCs hold the potential to differentiate into airway epithelial cells to ease ciliated cell loss in asthma [85]. As BMMSCs lack major histocompatibility complex (MHC) class II to escape immune surveillance, it is highly possible that these cells were able to transfer activity in different environment; for example, the upregulated proinflammatory cytokines promote BMMSCs to secrete anti-inflammatory factors [86]. BMMSCs were also proved to express many cytokine receptors, such as stromal cell-derived factor-1 (SDF-1) and C-X-C chemokine receptor type 4 (CXCR4), to help migration into inflammatory sites [87].

Interestingly, Nemeth et al. pointed that IL-4 and IL-13 may bind to the IL-4 receptor on BMMSCs and to stimulate the TGF-*β* production and STAT6 pathway, and the secretion of TGF-*β* reduced the expression of IL-4 and IL-13 in turn; Treg cells also could be activated by BMMSCs and then to alleviate airway inflammation and hyperresponsiveness [88]. Systemic administration of human BMMSCs or murine BMMSCs both showed effective curative effects in mixed Th2/Th17 allergic asthma by improving the activity of antigen-specific CD4 T lymphocyte and alleviating airway inflammation and airway hyperresponsiveness [89]. Some trials also proved the safety of BMMSCs. A clinical trial of ischemic cardiomyopathy (ICM) (NCT01087996) expressed that transendocardial injection of allogeneic and autologous BMMSCs both showed certain curative effects without acute immunogenic reaction [90]. A phase I clinical trial (NCT02013700) of BMSCs-based treatment in idiopathic pulmonary fibrosis (IPF) conveyed that the injection administration of 2 × 108 18 cells/infusion seems safe, while 21 adverse events were reported, such as the common cold and bronchitis, and two MSC-treated patients died because of IPF progression [91].

### 4.2. BMMSC-Exosome Could Regulate the Pathological Process in Asthma

Further studies identified that the anti-inflammatory effects of MSC may be mainly mediated by MSC-derived soluble factors, such as EVs, and MSC-EVs may be more effective than the cells themselves [92]. In the experiments of repairing damaged neurons by MSCs, MSCs can be detected in mouse brain parenchyma for just a little, BMMSCs-derived exosomes were then considered as the critical role in treatment [93]. In the model of acute lung injury, it was clearly observed that systemic administration of MSCs was effective in decreasing inflammation and repairing lung injury while the distributed levels were below 5% at the 2nd postinjury day, which further proved the ability of BMMSCs to secrete paracrine cytokines [94]. Besides, BMMSC-derived exosomes could not only inhibit inflammatory progress but also downregulate cell apoptosis in lung injury via decreasing the expression of NF-*κ*B and HMGB1 [95].

Lung interstitial macrophages (IMs) widely exist throughout the lung, especially in the alveolar septa [96]. In the progression of asthma, lung IMs may be tightly related to TLR4/MyD88 pathway to produce IL-10 for anti-inflammation and to regulate Th2- and Th17-mediated inflammation [97]. Macrophages consist of about 70% of all immune cells in the lung; the two different phenotypes of macrophage function were different: the expression of classically activated (M1) was upregulated in asthma, which was closely related to proinflammatory cytokines in promoting inflammation, and the alternatively activated (M2) phenotypes had the opposite effect [98]. After intranasal injection of BMMSC-derived exosomes, the distribution ratio of IMs increased obviously in the lung and was expressed as dose-dependent; meanwhile, the expression of IL-10 was increased to help alleviate inflammation [80]. MSC-derived exosome also be proved to decrease inflammatory factors and transform macrophage into M2 phenotype in mouse model of asthma [99]. Dong et al. pointed out that MSC-Exo may modulate the NF-*κ*B and PI3K/AKT signaling pathway and inhibit the expression of tumor necrosis factor receptor-associated factor 1 (TRAF1) to promote M2 macrophage polarization and improve airway hyperresponsiveness in severe, steroid-resistant asthma (SSRA) [100].

**Figure 2:**
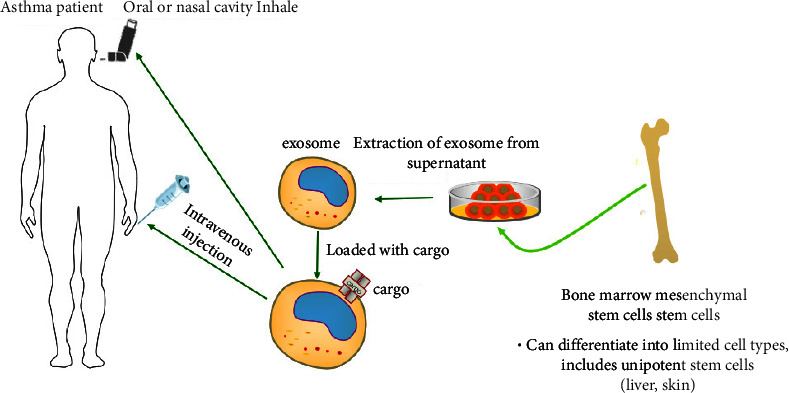
Purified exosomes which are derived from bone marrow mesenchymal stem cells, express various functions in treating asthma. Intravenous or inhalation administration both seem feasible methods for delivering exosomes, and the cargo carried by the exosomes could have important effects in treatment.

In addition, systemic administration of human BMMSCs either mouse BMMSCs-derived exosomes was effective in alleviating Th2/Th17-mediated airway inflammation, airway hyperresponsive, altering the phenotype of antigen-specific CD4 T cells in improving asthma [92]. BMMSC-Exo also played a certain role in upregulating the proliferation of Treg cells along with promoting functions in improving immune disorders, which may be modulated by CD14+ monocytes and CD19+ B cells; IL-10 and TGF-*β*1, as critical anti-inflammatory cytokines, also increased obviously compared with the model group [101]. It was acknowledged that Foxp3 was able to bind with the promoter of TGF-*β*1 to decrease T cell expression in asthma, and Foxp3 was also expressed by tolerogenic DCs in the lung [102]. Interestingly, it was proved that MSC-derived exosome played an important role in driving immature and mature DCs to differentiate into tolerogenic DCs, along with the higher levels of crucial and common anti-inflammatory cytokines, such as TGF-*β*1, IL-10, and the decrease of IL-6 [103]. In the model of allergic asthma rat, injecting BMMSCs-exosome could downregulate the expression of Th2 cytokines, inhibiting goblet cells proliferation and collagen deposition to reduce airway remodeling; and the Wnt/*β*-catenin pathway were considered as the main targeting signaling pathway [104]. The study about immune cells proliferation differentiation revealed that BMMSC-exosomes could suppress the functions of different immune cells, especially the B-lymphocytes, and the expression of mRNA showed significant different, which changed cells communications, cellular movements in the follows [105] (Figure 2).

## 5. Conclusion

Asthma is a common chronic respiratory disease with complex pathogenesis, multiple pathological types, and clinical phenotypes that influenced all age groups and causes a high burden on health care. Existing treatments benefit a lot despites some limitations. Exosome could carry with various proteins and RNAs and to modulate the cell-to-cell communications, influence biological process via delivering the contained cargo. BMMSC-derived exosomes caught researchers' eyes for their high safety, accurate targeting ability, high efficiency, and lack of side effects. Some experiments have found the potential of BMMSC-derived exosomes in modulating immune response, regulating macrophage polarization, inhibiting the expression of inflammatory factors and signaling pathways, and then inhibiting airway inflammation and remodeling in asthma. However, there are still some problems remained, such as limited economical and effective methods to industrially isolate and produce exosome, inadequate clinical evaluations in patients as most curative effect evaluations were limited in animal models. In conclusion, BMMSC-derived exosomes do hold various therapeutic potentials. It is highly possible to make full use of BMMSC-exosomes to benefit asthma patients.

## Figures and Tables

**Table 1:** Summary of applications of BMMSC-derived exosomes.

Diseases	Species	Functions	Reference
Asthma	Mice	Human bone marrow-derived MSCs (hBMSCs) could alter the phenotype of antigen-specific CD4 T cells, convey protective factors, and alleviate airway inflammation	[92] Cruz et al.
Diabetes	Mice	BMSC administration was able to reduce the diabetes-induced cognitive impairment and help repair damaged neurons, alleviate oxidative stress, and enhance synaptic numbers	[93] Nakano et al.
Acute lung injury	Mice	BMSCs injected could alleviate lung edema, inhibit proinflammatory response and enhance the production of IL-10 for anti-inflammation	[94] Gupta et al.
Smoke inhalation lung injury	Rats BEAS-2B and A549 cells	Exposure to exosomes successfully reversed the decrease in cell viability induced by smoke inhalation and alleviated inflammation and apoptosis in the rat lung. HMGB1/NF-*κ*B may act as main targets for BMSC-derived exosomes to improve lung injury	[95] Xu et al.
Asthma	Mice	Intranasal delivery of MSC-exosome could enhance IMs ratios in mouse lung and increase proinflammatory factors	[80] Ren et al.
Asthma	Mice	Systemic administration of human BMMSCs or mouse BMMSCs-derived exosomes was both effective in alleviating Th2/Th17-mediated airway inflammation, airway hyperresponsive, and altering the phenotype of antigen-specific CD4 T cells	[92] Cruz et al.
Asthma	Human bone marrow-derived mesenchymal stem cells, CD4+ CD25− T conv and CD4+ CD25+ Tregs	BMSC-exosome stimulates the expression of anti-inflammatory cytokines of PBMC, such as IL-10 and TGF-*β*; BMSC-exosome also showed potential in inducing Tregs polarization and suppressing immune which may be closely related with IL-10, TGF-*β*, and antigen presenting cells (APCs)	[101] Du et al.
Asthma	Rat BMMSC-derived exosomes	BMSCs-exosomes could inhibit the expression of Th2 cytokines, effectively suppress chronic allergic airway inflammation via Wnt/*β*-catenin pathway	[104] Song et al.

BMSCs, bone marrow mesenchymal stem cells; HMGB1, high mobility group protein; NF-*κ*B, nuclear factor kappa-B; PBMC, peripheral blood mononuclear cell; Tregs, regulatory cells; TGF-*β*, transforming growth factor-*β*; APC, antigen presenting cells.

## Data Availability

Not applicable.

## Abbreviations

ICS: Inhaled corticosteroid
LABA: Long-acting* β*-agonists
OCS: Oral corticosteroid
BMMSC: Bone marrow mesenchymal stem cell
EVs: Extracellular vesicles
SARP: Severe asthma research program
PEFR: Peak expiratory flow rate
FEV1: Forced expiratory volume in 1 second
EOS: Eosinophil
NE: Neutrophil
IgE: Immune globulin E
NETs: Neutrophil extracellular traps
PKC: Protein kinase C
NF-*κ*B: Nuclear factor kappa-B
I*κ*B: Inhibitory *κ*B proteins
LPS: Lipopolysaccharide
TLR4: Toll-like receptor 4
MyD88: Myeloid differentiation primary response 88
HMGB1: High mobility group box 1
HSF1: Heat shock factor 1
AHR: Airway hyperresponsiveness
TGF-*β*1: Transforming growth factor-*β*1
STAT: Signal transducers and activators of transcription
IL: Interleukin
ASMC: Airway smooth muscle cell
PTEN: Phosphatase and tensin homologue deleted on chromosome ten
CD38: Cluster of differentiation 38
CREB: Cyclic AMP response-element binding protein
TSLP: Thymic stromal lymphopoietin
PD: Panic disorder
MRT: Music and relaxation therapy
GCBT: Group cognitive behavioral therapy
SMA: Supplementary motor area
FC: Functional connectivity
TCM: Traditional Chinese medicine
SC: Scorpio and centipede
SAAT: Summer acupoint application treatment
MVBs: Multivesicular bodies
UTIs: Urinary tract infections
IBD: Inflammatory bowel disease
BALF: Bronchoalveolar lavage fluid
DCs: Dendritic cells
LTB4: Leukotrienes B4
HDM: House dust mite
miRNA: microRNA
MSCs: Mesenchymal stem cells
GVHD: Acute refractory graft-vs-host-disease
COPD: Chronic obstructive pulmonary disease
EPCs: Endothelial progenitor cells
SAEC: Small airway epithelial cells
OVA: Ovalbumin
MHC: Major histocompatibility complex
SDF-1: Stromal cell-derived factor-1
CXCR4: C-X-C chemokine receptor type 4
IPF: Idiopathic pulmonary fibrosis
IMs: Interstitial macrophages
TRAF1: Tumor necrosis factor receptor-associated factor 1
SSRA: Steroid-resistant asthma.
